# PDZ Binding Kinase/T-LAK Cell-Derived Protein Kinase Plays an Oncogenic Role and Promotes Immune Escape in Human Tumors

**DOI:** 10.1155/2021/8892479

**Published:** 2021-09-23

**Authors:** Tingting Feng, Yan Zhang, Sunbin Ling, Chenyang Xu, Yingqi Lyu, Tingting Lu, Xinyuan Liu, Lisha Ying, Yafeng Wan, Haijun Zhong, Dan Su

**Affiliations:** ^1^Department of Pathology, The Cancer Hospital of the University of Chinese Academy of Sciences (Zhejiang Cancer Hospital), Institute of Basic Medicine and Cancer (IBMC), Chinese Academy of Sciences, Hangzhou, Zhejiang 310022, China; ^2^Department of Colorectal Medicine, The Cancer Hospital of the University of Chinese Academy of Sciences (Zhejiang Cancer Hospital), Institute of Basic Medicine and Cancer (IBMC), Chinese Academy of Sciences, Hangzhou, Zhejiang 310022, China; ^3^Cancer Research Institute, The Cancer Hospital of the University of Chinese Academy of Sciences (Zhejiang Cancer Hospital), Institute of Basic Medicine and Cancer (IBMC), Chinese Academy of Sciences, Hangzhou, Zhejiang 310022, China; ^4^Department of Gerontology, The Second Affiliated Hospital of Zhejiang University School of Medicine, Hangzhou, Zhejiang 310009, China; ^5^Department of Hepatobiliary and Pancreatic Surgery, The Center for Integrated Oncology and Precision Medicine, Affiliated Hangzhou First People's Hospital, Zhejiang University School of Medicine, Hangzhou 310006, China

## Abstract

**Background:**

PDZ binding kinase (PBK)/T-LAK cell-derived protein kinase (TOPK) is an important mitotic kinase that promotes tumor progression in some cancers. However, the pan-cancer analysis of PBK/TOPK and its role in tumor immunity are limited.

**Methods:**

The oncogenic and immune roles of PBK in various cancers were explored using multiple databases, including Oncomine, Human Protein Atlas, ULCAN, Tumor Immune Estimation Resource 2.0, STRING, and Gene Expression Profiling Interactive Analysis 2, and data collected from The Cancer Genome Atlas and Genotype-Tissue Expression Project. Several bioinformatics tools and methods were used for quantitative analyses and panoramic descriptions, such as the DESeq2 and Tumor Immune Dysfunction and Exclusion (TIDE) algorithm.

**Results:**

PBK was expressed at higher levels in most solid tumors than in normal tissues in multiple databases. PBK was associated with an advanced tumor stage and grade and a poor prognosis in most cases. PBK was associated with tumor immune cell infiltration in most cases and was especially positively correlated with TAMs, Tregs, MDSCs, and T cell exhaustion in KIRC, LGG, and LIHC. PBK was closely related to TMB, MSI, and immune checkpoint genes in various cancers, and patients with higher expression of PBK in KIRC, LGG, and LIHC had higher TIDE scores and lower immune responses in the predicted results. PBK was closely related to cell cycle regulation and immune-related processes in LIHC and LGG according to GO and KEGG enrichment analyses.

**Conclusions:**

PBK may play an oncogenic role in most solid tumors and promotes immune escape, especially in KIRC, LGG, and LIHC. This study suggests the potential value of PBK inhibitors combined with immunotherapy.

## 1. Introduction

PDZ binding kinase (PBK) is a kinase that binds to the PDZ2 region of HDIG as first identified by an investigator screening a yeast two-hybrid system [1], and the gene was cloned from a HeLa cell cDNA library. T-LAK cell-derived protein kinase (TOPK) is a serine-threonine protein kinase identified from a cDNA library of activated lymphokine-activated killer T cells [2], and PBK was later identified as the same molecule as TOPK by a sequence analysis, hence receiving the name PBK/TOPK. PBK/TOPK is a potential target for antitumor therapy. First, based on the distribution of its expression, PBK/TOPK is rarely expressed in normal tissues, as its expression is detected only in testicular and embryonic tissues but is expressed at high levels in some malignancies; its expression level is negatively correlated with patient prognosis [3–5]. Second, regarding the molecular mechanism, PBK/TOPK, a mitotic kinase is involved in regulating multiple important signaling pathways such as p53 [6], MAPK [3], and PI3K/AKT [7], playing an important role in the process of tumorigenesis and tumor progression. Third, regarding the molecular properties, PBK/TOPK is a serine-threonine protein kinase, and several PBK/TOPK specific inhibitors have been screened, such as HI-TOPK-032, OTS514, and OTS964. In animal models, these inhibitors effectively inhibit the development of multiple malignant tumors with fewer toxic side effects [8–10], showing good clinical prospects.

Furthermore, several recent studies have suggested that PBK/TOPK may be associated with tumor-infiltrating immune cells and have a potential target for cancer immunotherapy [5, 11, 12]. This study is the first to conduct a pan-cancer analysis of PBK/TOPK using Oncomine, Human Protein Atlas(HPA), ULCAN, the Tumor Immune Estimation Resource 2.0 (TIMER2.0), STRING, Gene Expression Profiling Interactive Analysis 2 (GEPIA2), and the data collected from The Cancer Genome Atlas (TCGA) and Genotype-Tissue Expression Project (GTEx). The results of this study showed the potential correlation and mechanism of PBK/TOPK and tumor-immune interactions.

## 2. Materials and Methods

### 2.1. Analysis of PBK/TOPK Expression

Differences in PBK/TOPK mRNA expression across cancers in the Oncomine database (https://www.oncomine.org/) were analyzed. The thresholds were set as a fold change of 1.5 and *P* value of 0.001.

RNAseq data from TCGA and GTEx were downloaded from UCSC XENA (https://xenabrowser.net/datapages/) [13]. All RNAseq data were processed using the Toil process in TPM format. Notably, 33 cancers and 31 types of normal tissues (*N* = 15776) were included in the gene expression analysis. The difference in PBK/TOPK mRNA expression between tumors and paired adjacent noncancerous tissues from TCGA data was analyzed. Differences in PBK/TOPK mRNA expression between tumors and unpaired normal tissues from TCGA and GTEx data were also analyzed. The RNAseq data in TPM (transcripts per million reads) format were log2 transformed. If not stated otherwise, the analysis of “TCGA” in this study was based on the data downloaded from UCSC Xena.

Protein expression in 20 cancers (breast cancer, carcinoid, cervical cancer, colorectal cancer, endometrial cancer, glioma, head and neck cancer, liver cancer, lung cancer, lymphoma, melanoma, ovarian cancer, pancreatic cancer, prostate cancer, renal cancer, skin cancer, stomach cancer, testis cancer, thyroid cancer, and urothelial cancer) in the HPA database (https://www.proteinatlas.org/) was analyzed [14]. Images of immunohistochemical staining were available from the Human Protein Atlas version 20.1 (https://www.proteinatlas.org/ENSG00000168078-PBK/TOPK/pathology). Differences in PBK/TOPK protein levels were analyzed with the “CPTAC analysis” module in the UALCAN portal database (http://ualcan.path.uab.edu/) [15]. Differences in PBK/TOPK protein expression between tumors (breast cancer, ovarian cancer, colon cancer, clear cell renal cell carcinoma, uterine corpus endometrial carcinoma, lung adenocarcinoma, and pediatric brain cancer) and paired normal tissues were analyzed.

Cancers' name abbreviations were given as follows: adrenocortical carcinoma (ACC); Bladder Urothelial Carcinoma (BLCA); breast invasive carcinoma (BRCA); cervical squamous cell carcinoma and endocervical adenocarcinoma (CESC); cholangiocarcinoma (CHOL); colon adenocarcinoma (COAD); Lymphoid Neoplasm Diffuse Large B-cell Lymphoma (DLBCL); esophageal carcinoma (ESCA); glioblastoma multiforme (GBM); Head and Neck squamous cell carcinoma (HNSC); Kidney Chromophobe (KICH); kidney renal clear cell carcinoma (KIRC); kidney renal papillary cell carcinoma (KIRP); Acute Myeloid Leukemia (LAML); Brain Lower Grade Glioma (LGG); liver hepatocellular carcinoma (LIHC); lung adenocarcinoma (LUAD); Lung squamous cell carcinoma (LUSC); Mesothelioma (MESO); ovarian serous cystadenocarcinoma (OV); pancreatic adenocarcinoma (PAAD); Pheochromocytoma and Paraganglioma Prostate adenocarcinoma (PCPG); Prostate adenocarcinoma (PRAD); rectum adenocarcinoma (READ); Sarcoma (SARC); Skin Cutaneous Melanoma (SKCM); stomach adenocarcinoma (STAD); Testicular Germ Cell Tumors (TGCT); Thyroid carcinoma (THCA); Thymoma (THYM); Uterine Corpus Endometrial Carcinoma (UCEC); Uterine Carcinosarcoma (UCS); Uveal Melanoma (UVM); renal cell carcinoma (RCC).

### 2.2. Survival Analysis Based on PBK/TOPK Expression

The patients were stratified based on the expression of the PBK/TOPK mRNA in tumors and adjacent noncancerous tissues using a bipartite method and were divided into two groups: PBK/TOPK high expression and PBK/TOPK low expression groups. The correlation of PBK/TOPK expression with overall survival (OS) was analyzed with the Kaplan–Meier survival analysis.

### 2.3. Analysis of the Correlation of PBK/TOPK Expression with Immune Cell Infiltration

The immune cell infiltration score data were downloaded from the Timer2.0 database (http://timer.comp-genomics.org/) [16]. TIMER2.0 is a comprehensive resource for the systematical analysis of immune cell infiltrates and tumor immunological, clinical, and genomic features across diverse cancer types. “PBK/TOPK” was input in the “Gene” of the “Immune Association” module. The association of PBK/TOPK with selected immune cell types was analyzed. Timer2.0 provides six immune estimation algorithms, among which TIMER algorithms is the only one that considers tissue specificity. The associated data was downloaded from the “Table” module of TIMER2.0, and we present the TIMER algorithms-associated data of “T-cell CD8+,” “T-cell CD4+,” “B cell,” “Neutrophil,” “Macrophage,” and “Myeloid dendritic cell” across cancers as a heatmap with *R* software v4.0.3 and the “ComplexHeatmap” *R* package [17]. To further analyze the relationship of PBK with tumor-killing immune cells (CD8+ T cell and NK cell) as well as immune cells that facilitate tumor immune escape (M2 macrophage, Tregs, cancer-associated fibroblast (CAFs), and Myeloid-derived suppressor cells (MDSCs)) in KIRC, LGG, and LIHC, we displayed the images of the relevant data derived from the analysis based on the QUANTISEQ, MCP-COUNTER, and Tumor Immune Dysfunction and Exclusion (TIDE) algorithms in the TIMER2.0. The partial correlation (cor) values and *P* values were analyzed using Spearman's rank correlation test.

### 2.4. Analysis of the Correlation between PBK/TOPK Expression and Tumor Mutational Burden and Microsatellite Instability

TMB (tumor mutation burden) is derived from the article The Immune Landscape of Cancer published by Vesteinn Thorsson et al. in 2018 [18]. MSI (microsatellite instability) is derived from the Landscape of Microsatellite Instability Across 39 Cancer Types published by Russell Bonneville et al. in 2017 [19]. Spearman's rank correlation coefficients were calculated to analyze the correlations of PBK/TOPK expression with the TMB and the MSI of each tumor sample.

### 2.5. Analysis of the Correlation between PBK/TOPK Expression and the Immune Response

The association of PBK/TOPK expression with immune checkpoint gene expression in pan-cancers from TCGA was analyzed.

Potential immune checkpoint inhibitor (ICI) response was predicted with the TIDE algorithm [20, 21].

### 2.6. Analysis of the Correlation between PBK/TOPK Expression and DNA Mismatch Repair Genes and Methyltransferases

The association of PBK/TOPK expression with the expression of DNA mismatch repair gene (MLH1, MSH2, MSH6, and PMS2) and methyltransferases gene (DNMT1, DNMT3A, and DNMT3B) [22] was analyzed using the RNAseq data of 33 cancers from TCGA.

### 2.7. Gene Enrichment Analysis

STRING (https://www.string-db.org/) [23] and GEPIA2 (http://gepia2.cancer-pku.cn/) were used for the PBK/TOPK-associated enrichment analysis. “PBK/TOPK” was input into the “SEARCH” module, and “Homo sapiens” was subsequently selected. We next set the following parameters: network type (“full network”), meaning of network edges (“evidence”), active interaction source (“experiments”), minimum required interaction score (“medium confidence 0.150”), and max number of interactors to show (“no more than 10 interactors” in the 1st shell). Finally, the proteins binging to PBK/TOPK based on available experimental data were obtained.

The “Similar Gene Detection” module of GEPIA2 was used to obtain the top 100 PBK/TOPK-associated genes in TCGA tumors and normal tissues. Then, GEPIA2 “correlation analysis” module was used to analyze the Pearson correlation coefficients of PBK/TOPK with the top 100 selected genes. The results were visualized by constructing a dot plot. Then, we used TIMER2.0 to verify the correlation of PBK/TOPK expression with the five highest PBK/TOPK-correlated genes in GEPIA2. BIOINFORMATICS ＆ EVOLUTIONARY GENOMICS, a web viewer that calculates and draws Venn diagrams (http://bioinformatics.psb.ugent.be/webtools/Venn/), was used to prepare the Venn diagrams of the PBK/TOPK-binding and related genes. These selected genes and the differentially expressed genes between the PBK/TOPK high expression and low expression groups of KIRC, LGG, and LIHC in TCGA were subjected to KEGG and GO pathway enrichment analysis.

### 2.8. Statistical Analysis

All statistical analyses of the RNAseq data of TCGA were performed using *R* software v4.0.3. The Wilcoxon rank-sum test was used to analyze the expression differences between tumor and normal tissues. ANOVA was used for PBK/TOPK mRNA expression comparison between the different pathological stages. The Shapiro–Wilk normality test was used to determine the normal distribution of the data, and Levene's test was used for the homogeneity of variance analysis. One-way ANOVA was used if variances were homogeneous, and Welch's one-way ANOVA was used if variances were not homogeneous. The student's *t*-test was used for expression comparison of PBK/TOPK between different tumor grade groups. Independent samples *t*-tests were used if the data had a normal distribution, and Welch's *t*-tests were used if the data were not normally distributed. The correlation of PBK/TOPK expression and OS was analyzed with the Kaplan–Meier survival analysis. The “survival” *R* package was used for statistical analysis. Spearman's rank correlation coefficient was used to analyze the correlations of PBK/TOPK expression and TMB, MSI, immune checkpoint gene expression, DNA mismatch repair gene expression, and methyltransferase gene expression. The “clusterProfiler” was used for GO and KEGG enrichment analysis. And the “org.Hs.eg.db” *R* package (V.3.10.0) was used for ID transformation. We limit species to “*Homo sapiens*.” The thresholds were set as a fold change of 1.0 and *P* value of 0.05 in the TCGA datasets. The “ ggplot2” and “ggpubr” *R* packages were used to generate boxplots, violin plots, lollipop charts, and Bubble charts. The “forestplot” *R* package and “survminer” *R* package were used to generate forest plots and survival curves. The “ComplexHeatmap” was used to generate heatmaps. A *P* value <0.05 was considered statistically significant.

## 3. Results

### 3.1. PBK/TOPK Is Overexpressed in Various Cancers

**Figure 1:**
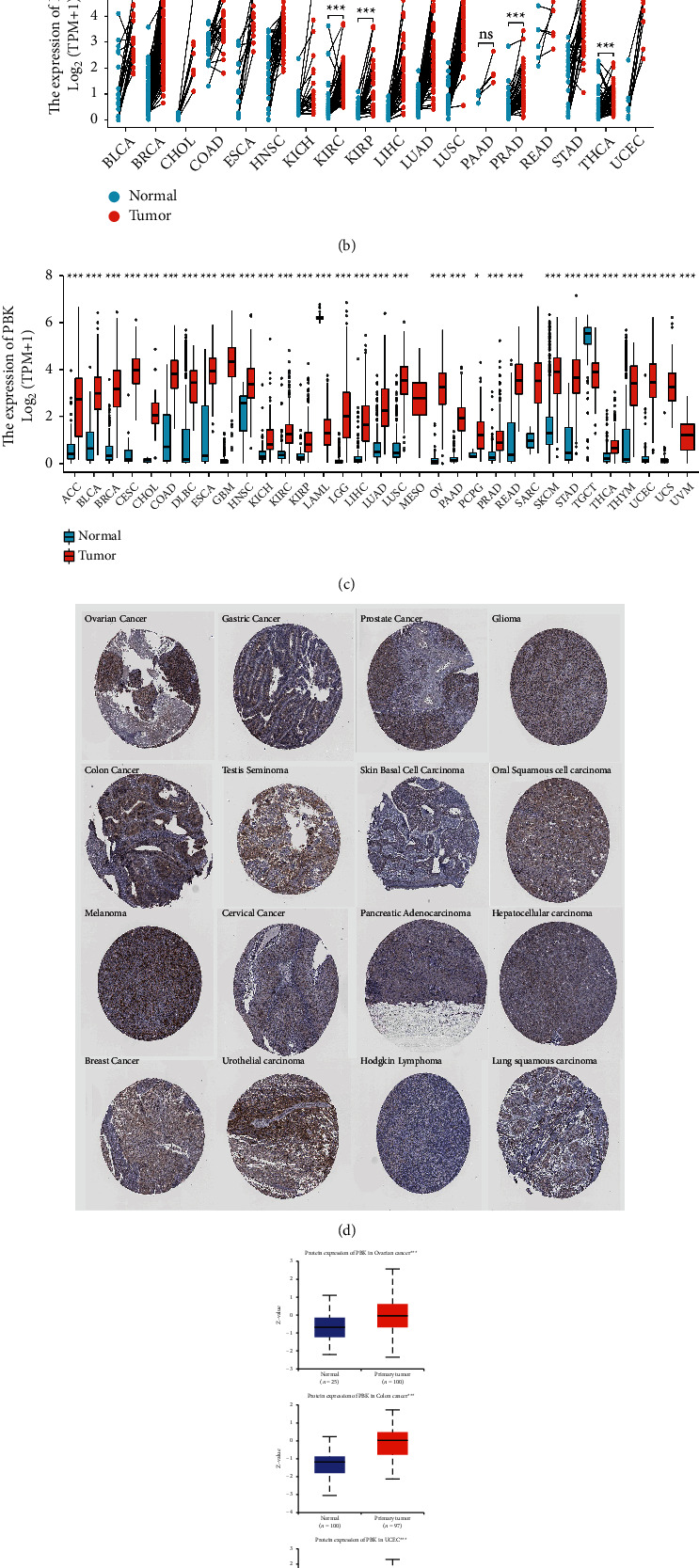
The expression of PBK in different types of cancers. (a) PBK mRNA expression in tumors compared to normal tissues in datasets from the Oncomine database (*N* = 74752). (b) Paired comparisons of differences in PBK mRNA expression levels between cancer and normal tissues in TCGA (*N* = 670). (c) Nonpaired comparisons of differences in PBK mRNA expression levels between cancer and normal tissues in TCGA + GTEx databases (*N* = 15776). (d) Image of PBK immunohistochemical staining in various cancers obtained from the Human Protein Atlas (*N* = 16). (e) Expression of the PBK protein in tumor tissues compared to the normal tissues from patients with various cancers in CPTAC datasets (^*∗*^*P* < 0.05,^*∗∗*^*P* < 0.01, and ^*∗∗∗*^*P* < 0.001) (*N* = 647).

The expression of PBK/TOPK mRNA and protein were explored. The PBK/TOPK mRNA was expressed at higher levels in tumors than in normal tissues in most tumors' datasets from Oncomine, such as bladder cancer, brain and CNS cancer, cervical cancer, colorectal cancer, esophageal cancer, gastric cancer, head and neck cancer, liver cancer, lung cancer, lymphoma, ovarian cancer, pancreatic cancer, prostate cancer, and sarcoma (Figure 1(a)). Meanwhile, lower expression of the PBK/TOPK mRNA was observed in the leukemia datasets. Notably, higher PBK/TOPK mRNA expression was found in 9 datasets, and lower PBK/TOPK mRNA expression was found in 1 dataset of breast cancer. Next, we analyzed PBK/TOPK mRNA expression in various cancers from TCGA. First, we performed paired comparisons of differences in PBK/TOPK mRNA expression levels between cancer and normal tissues from individuals with the same cancer. Only 18 tumors were eligible, and PBK/TOPK mRNA expression showed no significant differences only in PAAD and READ. Higher PBK/TOPK mRNA expression levels were detected in other tumors, such as BLCA, BRCA, CHOL, COAD, ESCA, HNSC, KICH, KIRC, KIRP, LIHC, LUAD, LUSC, PRAD, READ, STAD, THCA, and UCEC, than in tumor tissues compared to normal tissues (Figure 1(b); for additional details, see Table S1). Then, the normal tissues from GTEx were included. We reconfirmed the similar expression in the 18 cancers. Higher expression of the PBK/TOPK mRNA was detected in the following 12 cancers: ACC, CESC, DLBC, GBM, HNSC, LGG, OV, PAAD, PCPG, READ, SKCM, and THYM, while no significance was found in SARC. Because of the lack of matched normal tissues, MESO was not conducted statistical difference analysis (Figure 1(c); for additional details, see Table S1). Similar to the Oncomine results, lower expression of PBK/TOPK was observed in LAML tissues than that in normal tissues. Combining the aforementioned data, we found that the PBK/TOPK mRNA appeared to be expressed at high levels in 30 cancers, except LAML, SARC, and MESO.

The expression of the PBK/TOPK protein was obtained from the Human Protein Atlas and CPTAC datasets. Moderate nuclear and cytoplasmic positivity was observed in varying fractions of cells in several cases of ovarian, colorectal, melanoma, breast, stomach, testis, and cervical cancers. The remaining cancer tissues including urothelial carcinoma, prostate cancer, skin basal cell carcinoma, pancreatic adenocarcinoma, Hodgkin lymphoma, glioma, oral squamous carcinoma, hepatocellular carcinoma, and lung squamous carcinoma showed weak staining (Figure 1(d)). Endometrial cancer, renal cancer, carcinoid cancer, and thyroid cancer generally exhibited negative staining. We confirmed the high expression of the PBK/TOPK protein in COAD and OV in the CPTAC datasets. In addition, we also observed high expression of PBK/TOPK in UCEC and RCC tissues compared to the normal tissues in the CPTAC datasets (Figure 1(e)).

### 3.2. Analysis of PBK/TOPK mRNA Expression Based on the Pathological Stage and Tumor Grade

**Figure 2:**
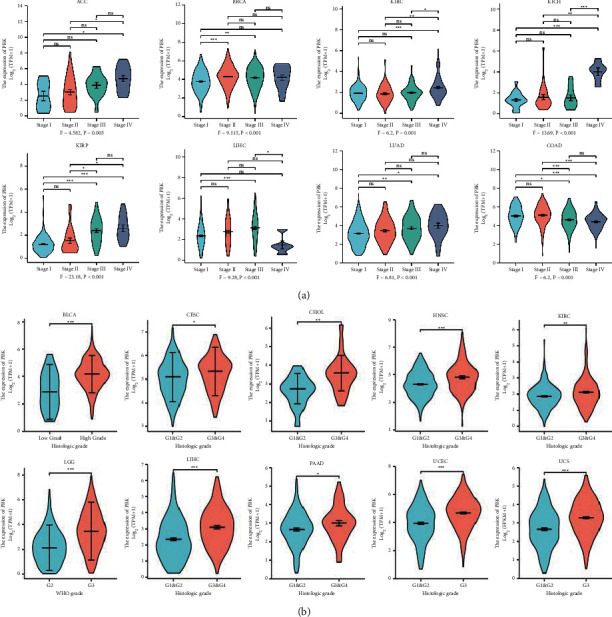
PBK mRNA expression based on the pathological stage and tumor grade of various cancers in TCGA. (a) PBK mRNA expression in cancers of different pathological stages (*N* = 7604). (b) PBK mRNA expression in the low-grade (G1 and G2) and high-grade groups of various cancers (^*∗*^*P* < 0.05,^*∗∗*^*P* < 0.01, and ^*∗∗∗*^*P* < 0.001) (*N* = 3372).

We explored PBK/TOPK mRNA expression in various tumors with different pathological stages in TCGA. We found that in the downloaded TCGA data, only 23 cancer datasets contained tumor staging information, while 12 cancer datasets contained histologic grade information. The expression of PBK/TOPK increased from pathologic stages I to IV in various cancers including ACC, KICH, KIRC, KIRP, and LUAD. The expression of PBK/TOPK increased in LIHC from stages I to II but was a lower expression in stage IV. Although elevated PBK expression in stages II and III compared to stage I was found in BRCA, no statistical difference was found in the comparison between the other staging groups. However, PBK/TOPK mRNA expression was negatively correlated with the pathological stage of COAD (Figure 2(a)). And no significance was found in 15 cancers including BLCA, CESC, CHOL, DLBC, ESCA, HNSC, LUSC, MESO, OV, READ, SKCM, TGCT, THCA, UCS, and UVM (Figures S1A–S10). In addition, we explored the correlation between the expression of the PBK/TOPK mRNA and the tumor grade. Higher expression of PBK/TOPK mRNA was found in high-grade (G3&G4) tumors than in low-grade (G1&G2) tumors of BLCA, CESE, CHOL, HNSC, KIRC, LGG, LIHC, PAAD, UCEC, and UCS (Figure 2(b)). And no significance was found in ESCA and OV (Figures S1P-S1Q).

### 3.3. PBK/TOPK Is Associated with Prognosis in Multiple Cancers

**Figure 3:**
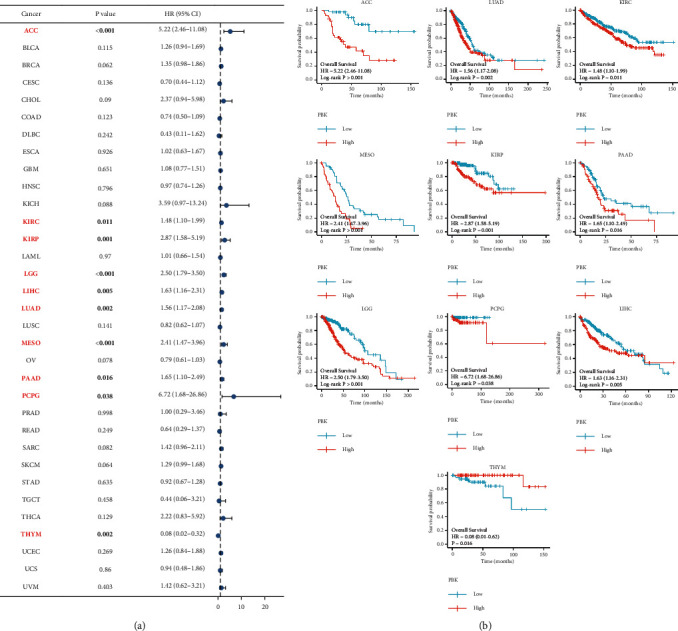
PBK mRNA expression and prognosis of patients with 33 cancers in TCGA. (a) Forest plot of the relationship between PBK mRNA expression and overall survival (OS) in months for patients with one of 33 tumors using the Kaplan–Meier analysis (*N* = 10363). (b) Kaplan–Meier OS curves for PBK mRNA expression in the 10 most significantly associated tumors (*N* = 3133).

In this study, we first explored the correlation of PBK/TOPK mRNA expression with 33 cancers in TCGA using the Kaplan–Meier survival analysis. As shown in Figure 3(a), the expression of PBK/TOPK was negatively correlated with the OS of patients with 9 tumors including, including ACC (HR = 5.22, log-rank *P* < 0.001), KIRC (HR = 1.48, log-rank *P*=0.011), KIRP (HR = 2.87, log-rank *P*=0.001), LGG (HR = 2.50, log-rank *P* < 0.001), LIHC (HR = 1.63, log-rank *P*=0.005), LUAD (HR = 1.56, log-rank *P*=0.002), MESO (HR = 2.41, log-rank *P* < 0.001), PAAD (HR = 1.65, log-rank *P*=0.016), and PCPG (HR = 6.72, log-rank *P*=0.038). Meanwhile, PBK/TOPK expression was found to play a protective role in THYM (HR = 0.08, log-rank *P*=0.002). The KM curves for the 10 tumors listed above are shown in Figure 3(b).

### 3.4. PBK/TOPK Is Correlated with Tumor Immune Cell Infiltration

**Figure 4:**
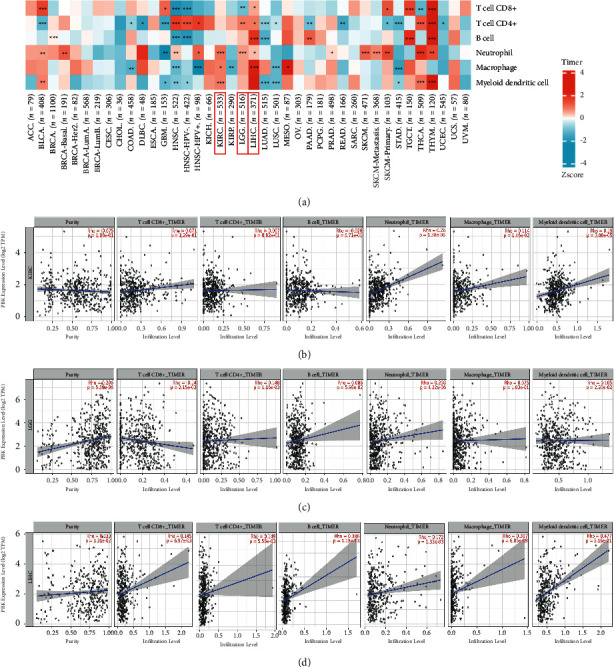
The correlations of PBK mRNA expression with tumor immune cell infiltration in pan-cancer using TIMER2.0. (a) Heatmap of the correlations between PBK expression and TILs determined using TIMER2.0. The Z-score method was used to standardize data. A significant correlation was found between PBK expression and TILs in most cancers (^*∗*^*P* < 0.05,^*∗∗*^*P* < 0.01, and ^*∗∗∗*^*P* < 0.001) (*N* = 12159). (b) Scatter plots of the correlations between PBK mRNA expression levels and immune infiltration cells (*N* = 1420).

Immune infiltration cells in the tumor microenvironment have been shown to play a key role in the occurrence and progression of cancer. Immune infiltration cells affect the effect of clinical treatments for cancer. A comprehensive analysis of immune infiltration cells will elucidate the mechanism of cancer immune escape, thus providing an opportunity to develop new therapeutic strategies [24]. Tumor purity was a major confounding factor in this analysis since most immune cell types were negatively correlated with tumor purity. As TIMER is the only algorithm that considers tissue specificity, we displayed the results of the correlation between PBK/TOPK and immune infiltration cells based on the TIMER algorithm of TIMER2.0. According to the data analyzed from the TIMER2.0, we redrew the heatmap (Figure 4(a)) to help readers more intuitively understand the correlation between PBK/TOPK and immune infiltration cells in pan-cancer datasets (Figure 4(a); for additional details, see Table S2). We observed a significant correlation between PBK/TOPK expression and TILs in most cancers. We focused on the 10 cancers in which PBK/TOPK affects prognosis. Finally, we focused on 3 tumors, KIRC, LGG, and LIHC, in which PBK/TOPK expression has a similar correlation with TILs. As shown in Figure 4(b), PBK/TOPK mRNA expression levels were significantly correlated with neutrophils (*R* = 0.26, *P* < 0.001), macrophages (*R* = 0.114, *P*=0.015), and DCs (*R* = 0.19, *P* < 0.001) in KIRC; CD8+ T cells (*R* = −0.14, *P*=0.002), CD4+ T cells (*R* = 0.148, *P*=0.001), neutrophils (*R* = 0.208, *P* < 0.001), and DCs (*R* = 0.105, *P*=0.022) in LGG; and CD8+ T cells (*R* = 0.145, *P*=0.006), CD4+ T cells (*R* = 0.149, *P*=0.006), B cells (*R* = 0.386, *P* < 0.001), neutrophils (*R* = 0.172, *P* = 0.001), macrophages (*R* = 0.307, *P* < 0.001), and DCs (*R* = 0.477, *P* < 0.001) in LIHC. Based on these results, PBK/TOPK may influence the prognosis of patients with cancer by modulating tumor immune cell infiltration.

### 3.5. Correlations of PBK mRNA Expression with the Infiltrating Immune Cells in KRIC, LGG, and LIHC Based on TIMER2.0

**Figure 5:**
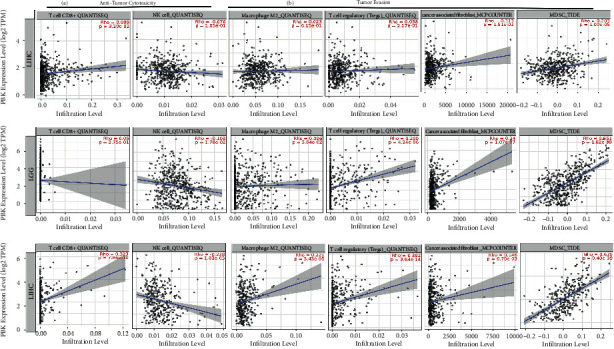
The relationship of PBK mRNA expression with the infiltrating immune cells in KRIC (*N* = 533), LGG (*N* = 516), and LIHC (*N* = 371) using TIMER2.0. (a) The correlation of PBK mRNA expression with CD8+ T cell and NK cell which may play a tumor-killing role in tumors. (b) The correlation of PBK mRNA expression with M2 macrophage, Tregs, CAFs, and MDSCs which play tumor evasion promotion roles in tumors.

We analyzed the correlations of PBK/TOPK expression with the infiltrating immune cells that exert tumor-killing effect (CD8+, NK cell) and promote tumor immune escape (M2 macrophage, Tregs, CAFs, MDSCs) based on TIMER2.0 to further explore the potential role of PBK in cancer immunity. As shown in Figure 5, similar to the results based on the TIMER algorithm, the relationships between PBK expression level and CD8+ T cell in 3 tumors were not consistent. In LIHC, PBK expression was positively correlated with CD8+ T cells (Rho = 0.323, *P* < 0.001) and KIRC (Rho = 0.099, *P*=0.0329) although the correlation coefficient was small in KIRC, and no significance was found in LGG (Rho = 0.05, *P*=0.275). PBK expression level was negatively correlated with NK cells in LGG (Rho = −0.108, *P*=0.018) and LIHC (Rho = −0.228, *P* < 0.001), and no significance was found in KIRC although a trend of negative correlation was found (Rho = −0.076, *P*=0.105). In LIHC, PBK expression level was significantly positively with M2 macrophage (Rho = 0.221, *P* < 0.001), Tregs (Rho = 0.392, *P* < 0.001), CAFs (Rho = 0.146, *P* < 0.01), and MDSCs (Rho = 0.625, *P* < 0.001). In LGG, PBK expression was significantly positively with M2 macrophage (Rho = 0.096, *P*=0.036), Tregs (Rho = 0.209, *P* < 0.001), CAFs (Rho = 0.24, *P* < 0.01), and MDSCs (Rho = 0.552, *P* < 0.001). In KIRC, PBK expression level was significantly positively with Tregs (Rho = 0.113, *P*=0.015) and MDSCs (Rho = 0.203, *P* < 0.001).

### 3.6. Correlations of PBK/TOPK Expression with the Biomarkers and the Immune Response for Immune Checkpoint Inhibitors

**Figure 6:**
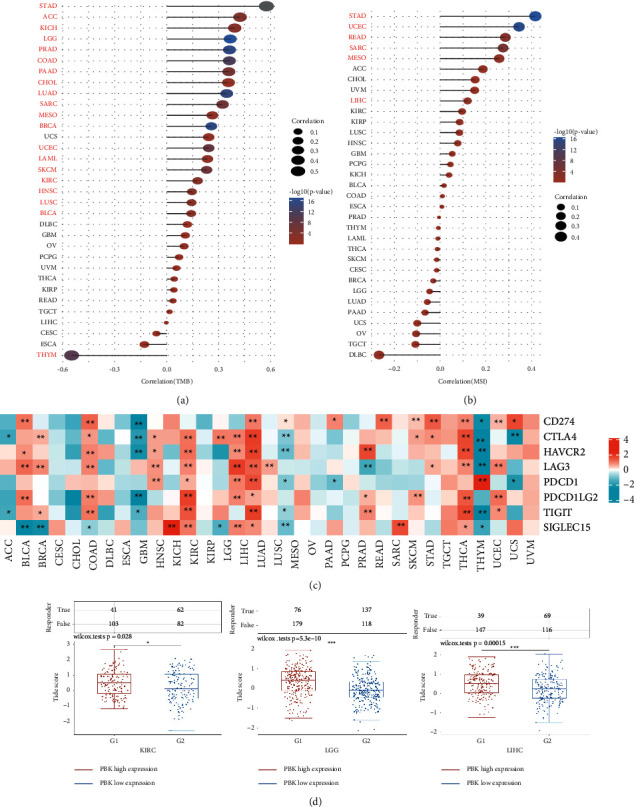
The individual correlations of PBK expression with TMB and MSI of each tumor type in TCGA (*N* = 10363). (a) The lollipop chart of the correlations of PBK expression with TMB (a) and MSI (b). The cancers marked in red indicate that the *P* value is less than 0.05. (c) Heatmap of correlations of PBK mRNA expression with the expression of different immune checkpoint genes in various cancers. (d) The numbers of immune responses and the distribution of TIDE scores for samples in the PBK high expression and low expression groups of KIRC (*N* = 288), LGG (*N* = 510), and LIHC (*N* = 371) in the predicted results (^*∗*^*P* < 0.05,^*∗∗*^*P* < 0.01, and ^*∗∗∗*^*P* < 0.001).

TMB is defined as the total number of detected somatic mutations per million bases. MSI is defined as the appearance of a new microsatellite allele at a locus due to the insertion or deletion of a repeat unit in a tumor compared with normal tissue. MSI occurs because of a functional defect in DNA mismatch repair in the tumor tissue. Both TMB and MSI are currently considered markers for the evaluation of the efficacy of immune checkpoint inhibitors therapy in a variety of tumors [25]. Patients with cancer presenting TMB-High or MSI may exhibit a better response to immunotherapy than those with TMB-Low or microsatellite stability (MSS). As shown in Figures 6(a) and 6(b), PBK/TOPK expression was significantly positively correlated with TMB in STAD, ACC, KICH, LGG, PRAD, COAD, PAAD, CHOL, LUAD, SARC, MESO, BRCA, UCEC, LAML, SKCM, KIRC, HNSC, LUSC, and BLCA and significantly negatively correlated in THYM. PBK/TOPK expression was significantly positively correlated with MSI in STAD, UCEC, READ, SARC, MESO, and LIHC (for additional details, see Tables S3 and S4).

Immune checkpoints are inhibitory pathways in the immune system that are regulated by ligand and receptor interactions. They are important for maintaining autoimmune tolerance and regulating the duration and amplitude of physiological immune responses to avoid the destruction and damage caused by immune cells to normal tissues. However, they may be exploited to participate in immune escape from cancer. The current immunotherapy drugs used in the clinic are mainly antibodies against these targets. PBK/TOPK expression showed different correlations with different immune checkpoint genes in various cancers (see Figure 6(c); for additional details, see Table S5). In KIRC, LGG, and LIHC, the expression of PBK/TOPK was positively correlated with the selected immune checkpoint genes. Furthermore, the potential ICI response was predicted with the TIDE algorithm. TIDE uses a set of gene expression markers to estimate 2 distinct mechanisms of tumor immune evasion, including dysfunction of tumor infiltration cytotoxic T lymphocytes (CTLs) and exclusion of CTLs by immunosuppressive factors. Patients with higher TIDE scores have a higher chance of antitumor immune escape, thus exhibiting a lower response rate to ICI treatment.  Figure 6(d) shows the numbers of immune responses and the distribution of TIDE scores of samples in the PBK/TOPK high expression and low expression groups of patients with KIRC, LGG, and LIHC in the predicted results. In all the 3 cancers, the PBK/TOPK overexpression group had a lower immune response rate and higher TIDE score in the predicted results (for additional details, see Table S6).

### 3.7. Correlations of PBK/TOPK Expression with DNA Mismatch Repair Genes and Methyltransferase Expression in Pan-Cancer

**Figure 7:**
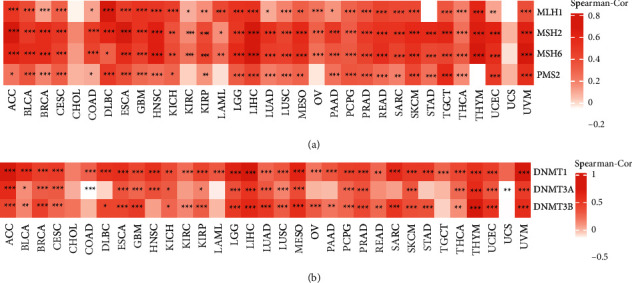
The correlations of PBK expression with the DNA mismatch repair gene (a) and methyltransferase (b) expression in pan-cancer data from TCGA (*N* = 10363). (a) The correlation of PBK expression with the expression of DNA mismatch repair genes in pan-cancer. (b) The correlation of PBK expression with methyltransferase expression in pan-cancer. Dark and light red colors represent different correlation coefficients (^*∗*^*P* < 0.05,^*∗∗*^*P* < 0.01, and ^*∗∗∗*^*P* < 0.001).

We further analyzed the correlation between PBK/TOPK expression and the expression of DNA mismatch repair genes and methyltransferase in pan-cancer. As shown in Figure 7(a), PBK/TOPK expression was positively correlated with the expression of DNA mismatch genes, including MLH1, MSH2, MSH6, and PMS2, in the majority of cancers except CHOL and UCS (see Figure 7(a); for additional details, see Table S7), suggesting that PBK/TOPK may promote cancer progression by upregulating DNA mismatch repair-related genes. In addition, DNA methylation may play an important role in tumor progression, and PBK/TOPK expression was positively correlated with methyltransferase expression, including DNMT1, DNMT3A, and DNMT3B, in most cancers except CHOL (see Figure 7(b); for additional details, see Table S7). This result suggested that PBK/TOPK potentially plays an important role in promoting tumorigenesis and progression by regulating gene methylation.

### 3.8. Enrichment Analysis of PBK/TOPK-Related Partners

**Figure 8:**
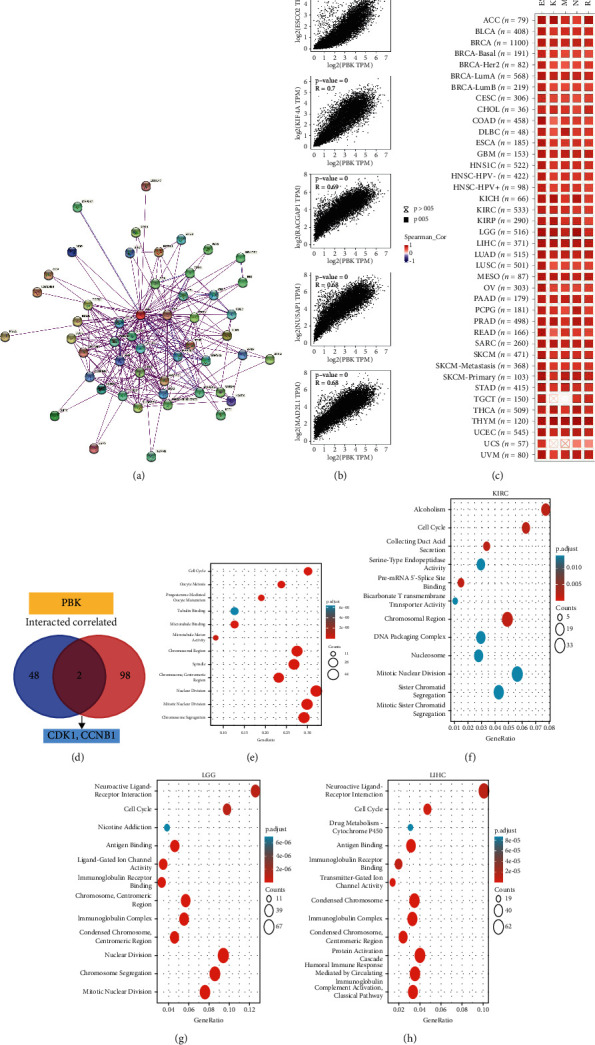
PBK-related pathway enrichment analysis. (a) The available experimentally determined PBK-binding proteins obtained using the STRING tool. (b) The top 100 PBK-correlated genes were obtained from GEPIA2. Scatter plots of the PBK mRNA expression with the selected targeting genes, including ESCO2, KIF4A, RACGAP1, NUSAP1, and MAD2L1. (c) Heatmap of PBK expression with the selected 5 genes in the specific cancers (*N* = 12159). (d) Venn diagram of the intersection analyses of 50 PBK-associated proteins and 100 PBK-correlated genes. (e) GO and KEGG pathway analyses of the PBK-binding and interacting genes. (f–h) GO and KEGG pathway analyses of PBK-related differentially genes in KIRC (f) (*N* = 288), LGG (g) (*N* = 510), and LIHC (h) (*N* = 371).

The target PBK/TOPK-binding proteins screened using STRING and PBK/TOPK expression-correlated genes screened using GEPIA2 were included in a series of enrichment analyses to further explore the molecular mechanism of the PBK/TOPK gene in tumorigenesis and tumor immunity. We obtained the top 50 PBK/TOPK-binding proteins based on experimental evidence in the STRING database and the top 100 PBK/TOPK-correlated genes in the pan-cancer dataset of GEPIA2. The correlations of PBK/TOPK with the top 5 correlated genes are shown in Figure 8(b), including ESCO2 (*R* = 0.74, *P* < 0.001), KIF4A (*R* = 0.7, *P* < 0.001), RACGAP1 (*R* = 0.69, *P* < 0.001), NUSAP1 (*R* = 0.68, *P* < 0.001), and MAD2L1 (*R* = 0.68, *P* < 0.001). As shown in the heatmap (see Figure 8(c); for additional details, see Table S9), PBK/TOPK also showed a positive correlation with these 5 genes in the majority of detailed cancer types. Then, we intersected 50 PBK/TOPK-associated proteins and 100 PBK/TOPK-correlated genes using the Venn diagram and obtained 2 coincident genes, CDK1 and CCNB1 (see Figure 8(d); for additional details, see Table S10). We further performed GO and KEGG pathway enrichment analyses for the above two datasets and found that PBK/TOPK was involved in cell cycle regulation such as “Cell Cycle,” “Oocyte Meiosis,” “Tubulin Binding,” “Microtubule Binding,” “Microtubule Motor Activity,” “Chromosomal Region,” “Spindle,” “Chromosome, Centromeric Region,” “Nuclear Division,” “Mitotic Nuclear Division,” “Chromosome Segregation,” and “Progesterone-Mediated Oocyte Maturation”.

We further performed GO and KEGG pathway enrichment analyses of PBK/TOPK-related differentially genes in KIRC, LGG, and LIHC. Similar to the results of the pan-cancer analysis, PBK/TOPK-related differentially expressed genes were enriched in cell cycle regulation. In addition, PBK/TOPK was involved in immune regulation processes such as “Antigen Binding,” “Immunoglobulin Receptor Binding,” and “Immunoglobulin Complex” in both LGG and LIHC. Moreover, PBK/TOPK-related differential genes of LIHC were also enriched in immune processes such as “humoral immune response mediated by circulation” and “complement Activation classic pathway” in LIHC (see in Figures 8(g) and 8(i), for additional details, see Tables S13 and S12). In KRIC, the PBK/TOPK-related differentially genes were enriched not only in cell cycle-related pathways but also in metabolism-related pathways such as “Alcoholism,” “Collecting Duct Acid secretion,” “Bicarbonate Transmembrane Transporter Activity,” and other processes such as “Serine-Type Endopeptidase Activity” which may be associated with the complement system (see in Figure 8(f); for detailed data, see Table S11).

## 4. Discussion

Although previous studies [1, 26, 27] have reported higher expression of the PBK/TOPK mRNA and protein in tumor tissues than in adjacent noncancerous tissues from different tumors, this study was the first pan-cancer analysis of PBK/TOPK and enabled us to observe the expression across all cancers. We only detected lower PBK/TOPK mRNA expression in LAML tissues than in normal tissues, which has not been reported in previous studies, although PBK/TOPK may mediate cell proliferation and viability in promyelocyte cell lines [28]. In addition, PBK/TOPK is reported to be upregulated and phosphorylated in HTLV-1-transformed T-cell lines and ATLL-derived T-cell lines [8]. Based on the complex classification of leukemia, the expression and role of PBK in each type of leukemia must be further studied. Images of immunohistochemical staining for PBK in various cancers obtained from the HPA database revealed the low to moderate expression of the PBK protein in many tumors. In previous studies, PBK was expressed at high levels in both tumors in which PBK was positively stained in the HPA database and in the negatively stained tumors in the HPA database [29, 30], such as CCA, ESCC, and GBM. Notably, the expression of the PBK protein in melanoma has not been reported; however, moderate nuclear and cytoplasmic positivity was observed in the melanoma IHC images we obtained from the HPA database but requires further confirmation in the future.

As a mitotic kinase, PBK plays an important role in the process of tumorigenesis and tumor progression. Not surprisingly, PBK expression was positively correlated with a more advanced pathological stage or differentiation grade of tumors, with the exception of COAD. The correlation of PBK/TOPK protein expression with the tumor stage in COAD remains unclear. No correlation was reported in the study by Su [31], while Zlobec et al. reported that diffuse TOPK was linked to an advanced pT stage only in patients with hereditary colorectal cancer but not in patients with sporadic colorectal cancer [32], which should be explored in further studies. Furthermore, we confirmed that PBK was associated with a poor prognosis in many solid tumor patients although high expression of PBK seems to be a good prognostic factor for THYM. It is noteworthy that PBK/TOPK expression was significantly negatively correlated with TMB in THYM which is also in contrast to other cancers. Integrated proteomics result of PBK based on ProteomicsDB, MaxQB, and MOPED obtained from the GENECARD website (https://www.genecards.org/cgi-bin/carddisp.pl?gene=PBK) suggests that PBK is likely expressed in immune cells. Because the thymus is an immune organ, the role of PBK in THYM may be different from other cancers and it deserves further exploration. PBK/TOPK expression was positively correlated with the expression of DNA mismatch genes and methyltransferase expression, in the majority of cancers except CHOL and UCS. This difference may be due to different genetic backgrounds of different cancers. Current studies of PBK in CHOL and UCS are limited, and further studies are needed. Overall, we propose that PBK represents a potential therapeutic target in cancer therapy.

Cancer immunity cycle can be divided into three stages: elimination, equilibrium, and escape. Cancer cells can escape the monitoring, recognition, and attack of the immune system and continue to proliferate by modifying their own surface antigens, recruiting inhibitory immune cells and molecules, and modifying the tumor microenvironment [33]. In this study, PBK expression was found closely related to immune infiltration cells in various cancers, especially in KIRC, LGG, and LIHC. PBK expression was positively correlated not only with immune infiltration cells including M2 macrophage, Tregs, CAFs, and MDSCs but also with T-cell exhaustion, which are involved in tumor immune escape [24, 34]. Similarly, Wang et al. suggested PBK/MSL1/CD276 signaling axis, which may play an important role in immune evasion of nasopharyngeal carcinoma and may be targeted for cancer immunotherapy [12]. Huang et al. identified PBK as one of the 14 hub genes correlated with immune cell infiltration in LIHC [11]. They found that the expression of these hub genes was significantly positively correlated with Tregs, TFH cells, and M0 macrophages, consistent with our results, but negatively correlated with monocytes, which is different from our results. This difference may be due to the weight of PBK in the hub genes. Moreover, PBK expression was negatively correlated with NK cells in LGG and LIHC. However, we did not find a consistent result of the correlation between PBK and CD8+ T cell in KIRC and LGG based on the TIMER and QUANTISEQ algorithm of TIMER2.0. On the one hand, the correlations were too weak to reach statistical differences; on the other hand, it also suggests that we should perform different bioinformatics algorithms to improve the dependability of analyzed results. This result suggested a very interesting phenomenon in which PBK, a mitotic kinase, may promote the proliferation of immune infiltration cells to potentially enhance immune escape. Moreover, although, in LIHC, PBK showed a positive correlation with CD8+ T cells, it still cannot be excluded that these cells may contain bystander CD8+ T cells that are not tumor killing [35], which remains to be verified by further experiments. We observed significant positive correlations between PBK expression and the TMB in KIRC and LGG, as well as MSI in LIHC. PBK expression was positively correlated with the expression of immune checkpoint genes in all 3 tumors. Given the complexity of effectors of immune efficacy, the efficacy of immune checkpoint inhibitor treatment cannot be effectively predicted by a single biomarker. We used the TIDE algorithm to evaluate the association of the immune response with PBK expression in the 3 cancers. TIDE is a bioinformatic algorithm to model 2 distinct mechanisms of tumor immune escape. PBK overexpression groups presented a higher TIDE score, suggesting more T-cell dysfunction and elimination characteristics in the 3 tumors, which again suggested the important role of PBK in tumor immune escape.

In addition to the important role of PBK in cell cycle regulation, we confirmed that PBK was closely related to immune regulation especially in LIHC and LGG via the enrichment analysis. In fact, it has been reported that potent kinase inhibitors of CDK4/6 with the goal of slowing tumor growth have been observed as a secondary unanticipated effect: exhibited a change in the TME, and increased sensitivity to ICI relative to controls in certain tumors including breast cancer, pancreatic cancer, and melanoma in preclinical studies [33]. Moreover, enhanced cell cycle progression signatures were significantly enriched in immunologically “cold” tumors compared with “hot” tumors [33, 36]. Due to the oncogenic role of PBK to promote the cell cycle in most tumors and its possible role in promoting immune escape, especially in KIRC, LGG, and LIHC, this study provides broad research space and clinical application prospects for PBK inhibitors combined with immunotherapy.

This study is a bioinformatics and computational biology analysis based on multiple databases and TCGA data that provides a new perspective on immune combination therapy but requires extensive validation in the future.

## 5. Conclusion

This study analysis revealed that PBK plays not only the oncogenic role in most solid tumors but also the role in promoting immune escape especially in KIRC, LGG, and LIHC. This study provides a potential for PBK inhibitors combined with immunotherapy.

## Data Availability

The generated and analyzed datasets of the current research are available in UCSC XENA (https://xenabrowser.net/datapages/), Oncomine (https://www.oncomine.org/), Human Protein Atlas database (https://www.proteinatlas.org/), Timer2.0 database (http://timer.comp-genomics.org/), STRING (https://www.string-db.org), and GEPIA2 (http://gepia2.cancer-pku.cn/).

## Supplementary Materials

Supplementary Materials Table S1: expression comparison of PBK/TOPK expression in tumor and normal tissues across cancers from TCGA and GTEx. Table S2: the correlation of PBK/TOPK expression with immune cells in TIMER2.0. Table S3: the correlation of PBK/TOPK expression with TMB across cancers from TCGA. Table S4: the correlation of PBK/TOPK expression with MSI across cancers from TCGA. Table S5: the correlation of PBK/TOPK expression with the expression of immune checkpoints genes across cancers from TCGA. Table S6: analysis of the correlation between PBK/TOPK expression and the immune response based on TIDE in KRIC, LGG, and LIHC. Table S7: the correlations of PBK/TOPK with DNA mismatch genes and methyltransferases. Table S8: similar genes of PBK/TOPK from GEPIA2. Table S9: the correlation of PBK/TOPK with top 5 similar genes from GEPIA2. Table S10: the result of Venn. Table S11: the GO and KEGG enrichment analysis of PBK/TOPK-related differentially genes in KIRC. Table S12: the GO and KEGG enrichment analysis of PBK/TOPK-related differentially genes in LGG. Table S13: the GO and KEGG enrichment analysis of PBK/TOPK-related differentially genes in LIHC. Table S14: Gene_outcome of PBK in the TIMER2.0 database. Figure S1: PBK mRNA expression based on the pathological stage and tumor grade of other cancers in TCGA. Click the link to download the supplements: (https://pan.baidu.com/s/1GFqYHhkAK0Y_34zLnH049g) (password 1234).
