# Supplementary material for: PDZ Binding Kinase/T-LAK Cell-Derived Protein Kinase Plays an Oncogenic Role and Promotes Immune Escape in Human Tumors
Source: J Oncol. 2021 Sep 23;2021:8892479. doi: 10.1155/2021/8892479 (PMC8486520; doi:10.1155/2021/8892479)
Supplement: Supplementary Materials — Table S1: expression comparison of PBK/TOPK expression in tumor and normal tissues across cancers from TCGA and GTEx. Table S2: the correlation of PBK/TOPK expression with immune cells in TIMER2.0. Table S3: the correlation of PBK/TOPK expression with TMB across cancers from TCGA. Table S4: the correlation of PBK/TOPK expression with MSI across cancers from TCGA. Table S5: the correlation of PBK/TOPK expression with the expression of immune checkpoints genes across cancers from TCGA. Table S6: analysis of the correlation between PBK/TOPK expression and the immune response based on TIDE in KRIC, LGG, and LIHC. Table S7: the correlations of PBK/TOPK with DNA mismatch genes and methyltransferases. Table S8: similar genes of PBK/TOPK from GEPIA2. Table S9: the correlation of PBK/TOPK with top 5 similar genes from GEPIA2. Table S10: the result of Venn. Table S11: the GO and KEGG enrichment analysis of PBK/TOPK-related differentially genes in KIRC. Table S12: the GO and KEGG enrichment analysis of PBK/TOPK-related differentially genes in LGG. Table S13: the GO and KEGG enrichment analysis of PBK/TOPK-related differentially genes in LIHC. Table S14: Gene_outcome of PBK in the TIMER2.0 database. Figure S1: PBK mRNA expression based on the pathological stage and tumor grade of other cancers in TCGA. Click the link to download the supplements: (https://pan.baidu.com/s/1GFqYHhkAK0Y_34zLnH049g) (password 1234). [file 8892479.f1.zip › 8892479.f1/Table S11.docx]

| ONTOLOGY | ID | Description | GeneRatio | BgRatio | pvalue | p.adjust | qvalue |
| --- | --- | --- | --- | --- | --- | --- | --- |
| BP | GO:0140014 | mitotic nuclear division | 33/585 | 264/18670 | 1.30e-11 | 4.76e-08 | 4.65e-08 |
| BP | GO:0000070 | mitotic sister chromatid segregation | 23/585 | 151/18670 | 3.41e-10 | 6.23e-07 | 6.09e-07 |
| BP | GO:0000819 | sister chromatid segregation | 25/585 | 189/18670 | 1.24e-09 | 1.51e-06 | 1.48e-06 |
| BP | GO:0000280 | nuclear division | 37/585 | 407/18670 | 7.14e-09 | 6.53e-06 | 6.38e-06 |
| BP | GO:0007059 | chromosome segregation | 31/585 | 321/18670 | 3.05e-08 | 2.23e-05 | 2.18e-05 |
| CC | GO:0044815 | DNA packaging complex | 18/611 | 115/19717 | 1.57e-08 | 6.67e-06 | 6.19e-06 |
| CC | GO:0000786 | nucleosome | 17/611 | 107/19717 | 3.13e-08 | 6.67e-06 | 6.19e-06 |
| CC | GO:0098687 | chromosomal region | 30/611 | 349/19717 | 5.17e-07 | 7.36e-05 | 6.83e-05 |
| CC | GO:0032993 | protein-DNA complex | 21/611 | 202/19717 | 1.35e-06 | 1.44e-04 | 1.33e-04 |
| CC | GO:0000788 | nuclear nucleosome | 9/611 | 38/19717 | 1.81e-06 | 1.49e-04 | 1.38e-04 |
| MF | GO:0030627 | pre-mRNA 5'-splice site binding | 7/475 | 24/17697 | 2.24e-06 | 0.001 | 0.001 |
| MF | GO:0004252 | serine-type endopeptidase activity | 14/475 | 160/17697 | 1.07e-04 | 0.014 | 0.013 |
| MF | GO:0015106 | bicarbonate transmembrane transporter activity | 5/475 | 19/17697 | 1.16e-04 | 0.014 | 0.013 |
| MF | GO:0008236 | serine-type peptidase activity | 15/475 | 182/17697 | 1.21e-04 | 0.014 | 0.013 |
| MF | GO:0030506 | ankyrin binding | 5/475 | 20/17697 | 1.51e-04 | 0.014 | 0.013 |
| KEGG | hsa04966 | Collecting duct acid secretion | 7/207 | 27/8076 | 3.77e-06 | 8.79e-04 | 8.34e-04 |
| KEGG | hsa04110 | Cell cycle | 13/207 | 124/8076 | 1.59e-05 | 0.002 | 0.002 |
| KEGG | hsa05034 | Alcoholism | 16/207 | 187/8076 | 2.25e-05 | 0.002 | 0.002 |
| KEGG | hsa05322 | Systemic lupus erythematosus | 13/207 | 136/8076 | 4.27e-05 | 0.002 | 0.002 |
| KEGG | hsa04721 | Synaptic vesicle cycle | 8/207 | 78/8076 | 8.18e-04 | 0.038 | 0.036 |

Table S11 The GO and KEGG enrichment analysis in KIRC.
