# Supplementary material for: PDZ Binding Kinase/T-LAK Cell-Derived Protein Kinase Plays an Oncogenic Role and Promotes Immune Escape in Human Tumors
Source: J Oncol. 2021 Sep 23;2021:8892479. doi: 10.1155/2021/8892479 (PMC8486520; doi:10.1155/2021/8892479)
Supplement: Supplementary Materials — Table S1: expression comparison of PBK/TOPK expression in tumor and normal tissues across cancers from TCGA and GTEx. Table S2: the correlation of PBK/TOPK expression with immune cells in TIMER2.0. Table S3: the correlation of PBK/TOPK expression with TMB across cancers from TCGA. Table S4: the correlation of PBK/TOPK expression with MSI across cancers from TCGA. Table S5: the correlation of PBK/TOPK expression with the expression of immune checkpoints genes across cancers from TCGA. Table S6: analysis of the correlation between PBK/TOPK expression and the immune response based on TIDE in KRIC, LGG, and LIHC. Table S7: the correlations of PBK/TOPK with DNA mismatch genes and methyltransferases. Table S8: similar genes of PBK/TOPK from GEPIA2. Table S9: the correlation of PBK/TOPK with top 5 similar genes from GEPIA2. Table S10: the result of Venn. Table S11: the GO and KEGG enrichment analysis of PBK/TOPK-related differentially genes in KIRC. Table S12: the GO and KEGG enrichment analysis of PBK/TOPK-related differentially genes in LGG. Table S13: the GO and KEGG enrichment analysis of PBK/TOPK-related differentially genes in LIHC. Table S14: Gene_outcome of PBK in the TIMER2.0 database. Figure S1: PBK mRNA expression based on the pathological stage and tumor grade of other cancers in TCGA. Click the link to download the supplements: (https://pan.baidu.com/s/1GFqYHhkAK0Y_34zLnH049g) (password 1234). [file 8892479.f1.zip › 8892479.f1/Table S12.docx]

Table S12 The GO and KEGG enrichment analysis in LGG.

| ONTOLOGY | ID | Description | GeneRatio | BgRatio | pvalue | p.adjust | qvalue |
| --- | --- | --- | --- | --- | --- | --- | --- |
| BP | GO:0007059 | chromosome segregation | 61/711 | 321/18670 | 7.73e-26 | 3.27e-22 | 2.82e-22 |
| BP | GO:0140014 | mitotic nuclear division | 54/711 | 264/18670 | 1.37e-24 | 1.91e-21 | 1.65e-21 |
| BP | GO:0000280 | nuclear division | 67/711 | 407/18670 | 1.52e-24 | 1.91e-21 | 1.65e-21 |
| BP | GO:0000819 | sister chromatid segregation | 46/711 | 189/18670 | 1.81e-24 | 1.91e-21 | 1.65e-21 |
| BP | GO:0000070 | mitotic sister chromatid segregation | 41/711 | 151/18670 | 6.58e-24 | 5.57e-21 | 4.80e-21 |
| CC | GO:0019814 | immunoglobulin complex | 41/739 | 159/19717 | 3.30e-23 | 1.53e-20 | 1.24e-20 |
| CC | GO:0000779 | condensed chromosome, centromeric region | 34/739 | 118/19717 | 3.75e-21 | 8.70e-19 | 7.05e-19 |
| CC | GO:0000775 | chromosome, centromeric region | 42/739 | 193/19717 | 1.10e-20 | 1.70e-18 | 1.38e-18 |
| CC | GO:0000776 | kinetochore | 35/739 | 135/19717 | 4.45e-20 | 5.16e-18 | 4.18e-18 |
| CC | GO:0000793 | condensed chromosome | 44/739 | 223/19717 | 7.69e-20 | 7.14e-18 | 5.78e-18 |
| MF | GO:0034987 | immunoglobulin receptor binding | 23/693 | 76/17697 | 7.27e-15 | 5.00e-12 | 4.44e-12 |
| MF | GO:0003823 | antigen binding | 32/693 | 160/17697 | 1.89e-14 | 6.52e-12 | 5.78e-12 |
| MF | GO:0015276 | ligand-gated ion channel activity | 24/693 | 138/17697 | 7.42e-10 | 1.28e-07 | 1.13e-07 |
| MF | GO:0022834 | ligand-gated channel activity | 24/693 | 138/17697 | 7.42e-10 | 1.28e-07 | 1.13e-07 |
| MF | GO:0022839 | ion gated channel activity | 39/693 | 334/17697 | 1.12e-09 | 1.54e-07 | 1.37e-07 |
| KEGG | hsa04110 | Cell cycle | 28/286 | 124/8076 | 1.53e-15 | 3.77e-13 | 3.24e-13 |
| KEGG | hsa04080 | Neuroactive ligand-receptor interaction | 36/286 | 341/8076 | 2.84e-09 | 3.49e-07 | 3.00e-07 |
| KEGG | hsa05033 | Nicotine addiction | 11/286 | 40/8076 | 8.39e-08 | 6.88e-06 | 5.92e-06 |
| KEGG | hsa04114 | Oocyte meiosis | 19/286 | 129/8076 | 1.14e-07 | 7.03e-06 | 6.05e-06 |
| KEGG | hsa03460 | Fanconi anemia pathway | 10/286 | 54/8076 | 1.59e-05 | 7.84e-04 | 6.74e-04 |
