# Supplementary material for: PDZ Binding Kinase/T-LAK Cell-Derived Protein Kinase Plays an Oncogenic Role and Promotes Immune Escape in Human Tumors
Source: J Oncol. 2021 Sep 23;2021:8892479. doi: 10.1155/2021/8892479 (PMC8486520; doi:10.1155/2021/8892479)
Supplement: Supplementary Materials — Table S1: expression comparison of PBK/TOPK expression in tumor and normal tissues across cancers from TCGA and GTEx. Table S2: the correlation of PBK/TOPK expression with immune cells in TIMER2.0. Table S3: the correlation of PBK/TOPK expression with TMB across cancers from TCGA. Table S4: the correlation of PBK/TOPK expression with MSI across cancers from TCGA. Table S5: the correlation of PBK/TOPK expression with the expression of immune checkpoints genes across cancers from TCGA. Table S6: analysis of the correlation between PBK/TOPK expression and the immune response based on TIDE in KRIC, LGG, and LIHC. Table S7: the correlations of PBK/TOPK with DNA mismatch genes and methyltransferases. Table S8: similar genes of PBK/TOPK from GEPIA2. Table S9: the correlation of PBK/TOPK with top 5 similar genes from GEPIA2. Table S10: the result of Venn. Table S11: the GO and KEGG enrichment analysis of PBK/TOPK-related differentially genes in KIRC. Table S12: the GO and KEGG enrichment analysis of PBK/TOPK-related differentially genes in LGG. Table S13: the GO and KEGG enrichment analysis of PBK/TOPK-related differentially genes in LIHC. Table S14: Gene_outcome of PBK in the TIMER2.0 database. Figure S1: PBK mRNA expression based on the pathological stage and tumor grade of other cancers in TCGA. Click the link to download the supplements: (https://pan.baidu.com/s/1GFqYHhkAK0Y_34zLnH049g) (password 1234). [file 8892479.f1.zip › 8892479.f1/Table S13.docx]

| ONTOLOGY | ID | Description | GeneRatio | BgRatio | pvalue | p.adjust | qvalue |
| --- | --- | --- | --- | --- | --- | --- | --- |
| BP | GO:0002455 | humoral immune response mediated by circulating immunoglobulin | 53/1504 | 150/18670 | 2.75e-21 | 1.44e-17 | 1.33e-17 |
| BP | GO:0006958 | complement activation, classical pathway | 50/1504 | 137/18670 | 7.20e-21 | 1.89e-17 | 1.74e-17 |
| BP | GO:0072376 | protein activation cascade | 60/1504 | 198/18670 | 4.56e-20 | 6.06e-17 | 5.58e-17 |
| BP | GO:0006956 | complement activation | 56/1504 | 175/18670 | 4.63e-20 | 6.06e-17 | 5.58e-17 |
| BP | GO:0000280 | nuclear division | 89/1504 | 407/18670 | 1.63e-18 | 1.71e-15 | 1.57e-15 |
| CC | GO:0019814 | immunoglobulin complex | 52/1591 | 159/19717 | 3.77e-19 | 2.16e-16 | 1.86e-16 |
| CC | GO:0000793 | condensed chromosome | 55/1591 | 223/19717 | 3.60e-14 | 7.63e-12 | 6.56e-12 |
| CC | GO:0000779 | condensed chromosome, centromeric region | 38/1591 | 118/19717 | 3.99e-14 | 7.63e-12 | 6.56e-12 |
| CC | GO:0000775 | chromosome, centromeric region | 49/1591 | 193/19717 | 2.76e-13 | 3.95e-11 | 3.40e-11 |
| CC | GO:0042571 | immunoglobulin complex, circulating | 28/1591 | 72/19717 | 4.49e-13 | 5.15e-11 | 4.43e-11 |
| MF | GO:0003823 | antigen binding | 45/1434 | 160/17697 | 5.46e-14 | 5.03e-11 | 4.44e-11 |
| MF | GO:0034987 | immunoglobulin receptor binding | 28/1434 | 76/17697 | 2.34e-12 | 1.08e-09 | 9.51e-10 |
| MF | GO:0022824 | transmitter-gated ion channel activity | 20/1434 | 61/17697 | 3.22e-08 | 7.43e-06 | 6.55e-06 |
| MF | GO:0022835 | transmitter-gated channel activity | 20/1434 | 61/17697 | 3.22e-08 | 7.43e-06 | 6.55e-06 |
| MF | GO:0030594 | neurotransmitter receptor activity | 28/1434 | 117/17697 | 1.42e-07 | 2.05e-05 | 1.81e-05 |
| KEGG | hsa04080 | Neuroactive ligand-receptor interaction | 62/615 | 341/8076 | 5.06e-11 | 1.53e-08 | 1.36e-08 |
| KEGG | hsa04110 | Cell cycle | 29/615 | 124/8076 | 3.18e-08 | 4.80e-06 | 4.28e-06 |
| KEGG | hsa00982 | Drug metabolism - cytochrome P450 | 19/615 | 71/8076 | 8.62e-07 | 8.68e-05 | 7.75e-05 |
| KEGG | hsa00830 | Retinol metabolism | 17/615 | 68/8076 | 8.88e-06 | 6.71e-04 | 5.99e-04 |
| KEGG | hsa05033 | Nicotine addiction | 12/615 | 40/8076 | 2.62e-05 | 0.001 | 0.001 |

Table S13 The GO and KEGG enrichment analysis in LIHC.
