# Supplementary figures and images for: PDZ Binding Kinase/T-LAK Cell-Derived Protein Kinase Plays an Oncogenic Role and Promotes Immune Escape in Human Tumors
Source: J Oncol. 2021 Sep 23;2021:8892479. doi: 10.1155/2021/8892479 (PMC8486520; doi:10.1155/2021/8892479)

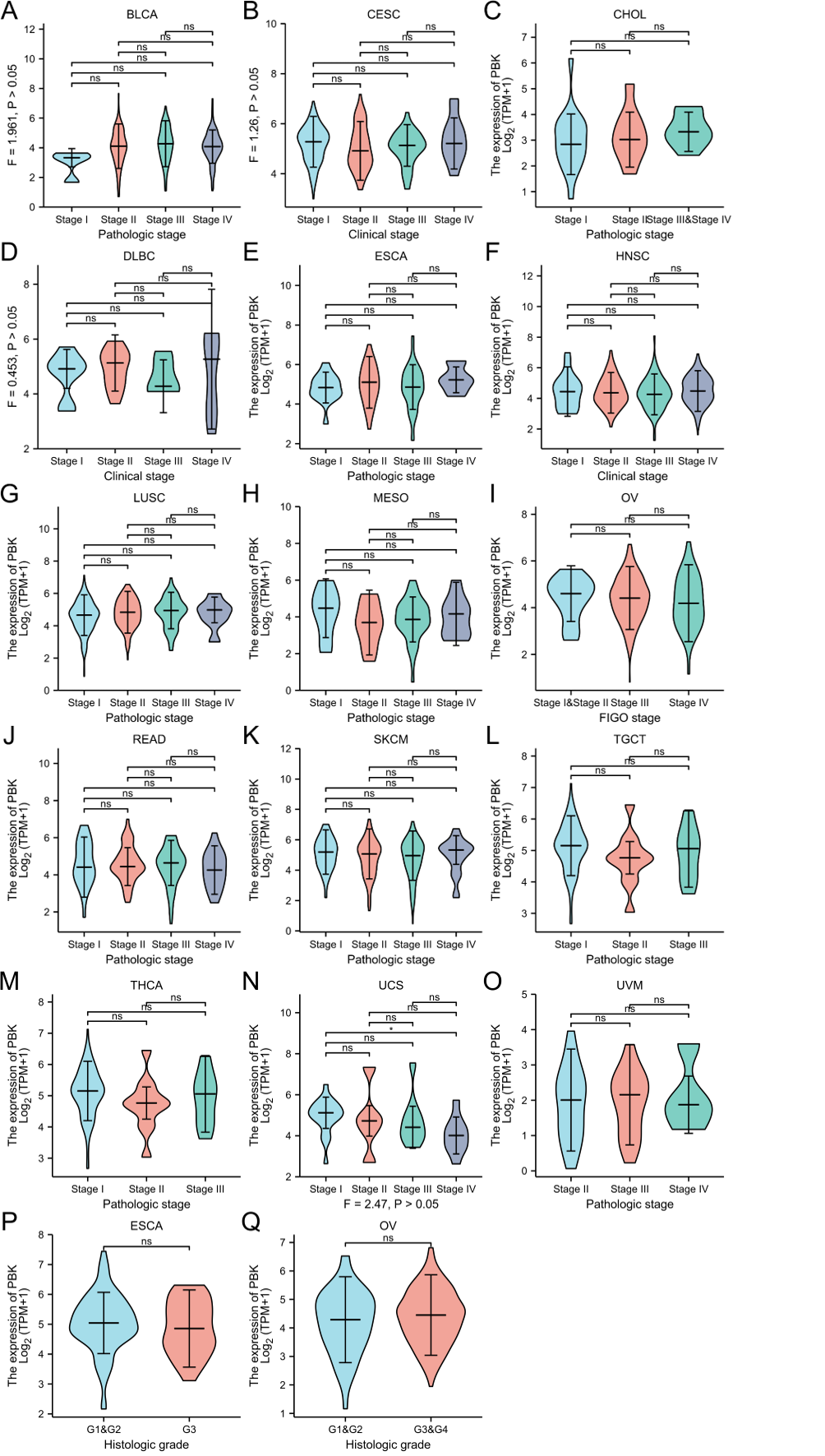

Supplement: Supplementary Materials — Table S1: expression comparison of PBK/TOPK expression in tumor and normal tissues across cancers from TCGA and GTEx. Table S2: the correlation of PBK/TOPK expression with immune cells in TIMER2.0. Table S3: the correlation of PBK/TOPK expression with TMB across cancers from TCGA. Table S4: the correlation of PBK/TOPK expression with MSI across cancers from TCGA. Table S5: the correlation of PBK/TOPK expression with the expression of immune checkpoints genes across cancers from TCGA. Table S6: analysis of the correlation between PBK/TOPK expression and the immune response based on TIDE in KRIC, LGG, and LIHC. Table S7: the correlations of PBK/TOPK with DNA mismatch genes and methyltransferases. Table S8: similar genes of PBK/TOPK from GEPIA2. Table S9: the correlation of PBK/TOPK with top 5 similar genes from GEPIA2. Table S10: the result of Venn. Table S11: the GO and KEGG enrichment analysis of PBK/TOPK-related differentially genes in KIRC. Table S12: the GO and KEGG enrichment analysis of PBK/TOPK-related differentially genes in LGG. Table S13: the GO and KEGG enrichment analysis of PBK/TOPK-related differentially genes in LIHC. Table S14: Gene_outcome of PBK in the TIMER2.0 database. Figure S1: PBK mRNA expression based on the pathological stage and tumor grade of other cancers in TCGA. Click the link to download the supplements: (https://pan.baidu.com/s/1GFqYHhkAK0Y_34zLnH049g) (password 1234). [file 8892479.f1.zip › 8892479.f1/Figure S1.docx]
